# Supplementary material for: Complexome profiling of the Chlamydomonas psb28 mutant reveals TEF5 as an early PSII assembly factor
Source: Plant Cell. 2025 Mar 17;37(6):koaf055. doi: 10.1093/plcell/koaf055 (PMC12164590; doi:10.1093/plcell/koaf055)
Supplement: koaf055_Supplementary_Data [file koaf055_supplementary_data.zip › psb28_interactive.zip!/plots/intensity/qualityPlots/peptides/RPSA_Cre10.g432800.t1.2.html]

### Cre10.g432800.t1.2

Trivial name: RPSA  
SeqCoverage [median]: 7.20  
Mass: 30.71 kDa  
  
 

| Sequence | MissedCleavage | Mass [Da] | wtIntensity | muIntensity | Score |
| --- | --- | --- | --- | --- | --- |
| HTPGTFTNQIQK | 0 | 1370.69 | 1794.76 | 0.00 | 155.97 |
| LLILTDPR | 0 | 939.58 | 3109.50 | 3545.91 | 114.60 |
|  |  |  | 4904.3 | 3545.9 |  |
